# Supplementary material for: Baseline assessment of knowledge, attitude, practice, and adherence toward antimicrobials among women living in two urban municipalities in Lalitpur district, Nepal
Source: PLoS One. 2025 Jan 9;20(1):e0317092. doi: 10.1371/journal.pone.0317092 (PMC11717222; doi:10.1371/journal.pone.0317092)
Supplement: S1 Table — (DOCX) [file pone.0317092.s001.docx]

Appendix 2: Health and health services access indictors of the two municipalities studied

| Indicators | Mahalaxmi | Godawari |
| --- | --- | --- |
| Female Population Over 18 Years | 85982 | 84177 |
| Disease Burden | Hypertension, Diabetes, Chronic Obstructive Pulmonary Disease (COPD), Asthama, Pneumonia, Acute Respiratory Infection  Gastritis  Upper Respiratory Tract Infection  Headache, Fever  Hypertension  Cough  Tonsilitis  Influenza | Diabetes, Gastritis, Acute Respiratory Tract Infection/Lower Respiratory Tract Infection, Upper Respiratory Tract Infection, Hypertension, Chronic Obstructive Pulmonary Disease (COPD), Headache, Toothache, Acute and Chronic Bronchitis |
| Healthcare Access | City Hospital =1,  Primary Healthcare Center = 2,  Health Post=10,  Ayurveda Dispensary =2,  Urban Health Center = 4,  Aadharbhut Swastha Sewa Kendra =2,  Birthing Center =5,  Leprosy Microscopy Center = 5,  Outpatient Therapeutic Center (OTC) Center = 5,  Directly Observed Treatment Short course (DOTS) Center, Drug Resistant Tuberculosis Sub Center = 16,  Prevention and Control of Mother to Child Transmission for HIV (PMTCT) Sites = 20  Kishor Kishori (Children) Clinic =5 | City Hospital (Private Hospital = 1 (KIST) Tertiary Hospital  Primary Healthcare Center = 1 (Luvu PHC)  Health Post = 4 (Imadol Health Post, Siddhipur Health Post, Tikathali Health Post, Lamatar Health Post)  Ayurveda Dispensary = 1(Nagarik Arogya Sewa Kendra (Health Center) Lamatar)  Urban Health Center = Not Available  Aadharbhut Swastha Sewa Kendra (Health Center) = 4, Basic Heath Services Center (BHSC) -2, Basic Heath Servics Center -3, Basic Heath Servics Center -4, Basic Heath Services Center -5, Basic Heath Services Center-9)  Birthing Center = 2 (Luvu Primary Healthcare Center & KIST)  Leprosy Microscopy Center = NA  Outpatient Therapeutic (OTC) Center = 1  Directly Observed Treatment Short course (DOTS) Center (DOTS) center = 11 Drug Resistant Tuberculosis Sub Center = NA  Prevention and Control of Mother to Child Transmission for HIV (PMTCT) Sites = 11  Kishor Kishori (Children) Clinic = 1(Lubhu PHC) |
| Number of FCHVs | 45 | 114 |
| Number of mothers under FCHVs | Not fixed, varies from 10 to 25 | Not fixed, varies from 10-25 |
