# Supplementary material for: Baseline assessment of knowledge, attitude, practice, and adherence toward antimicrobials among women living in two urban municipalities in Lalitpur district, Nepal
Source: PLoS One. 2025 Jan 9;20(1):e0317092. doi: 10.1371/journal.pone.0317092 (PMC11717222; doi:10.1371/journal.pone.0317092)
Supplement: S2 Table — (DOCX) [file pone.0317092.s002.docx]

Table 1: Socio demographic variables by municipalities, AMR baseline survey 2024. [n=1207]

| **Variable** | **median ± iqr** | **p-value** |
| --- | --- | --- |
| Age (in years) | | |
| Mahalaxmi (n=580) (n, %) | 45 ± 17 | 0.800 |
| Godawari (n=627) (n, %) | 42 ± 14 |  |
| Work experience in years | | |
| Mahalaxmi | 10 ± 17 | 0.526 |
| Godawari | 10 ± 14 |  |
| Number of household members | | |
| Mahalaxmi | 4 ± 2 | 0.491 |
| Godawari | 4 ± 1 |  |
| Number of household members with chronic disease | | |
| Mahalaxmi | 1 ± 0 | 0.762 |
| Godawari | 1 ± 1 |  |
| **Variable Yes (n, %) P value** | | |
| Presence of respiratory disease in the household | | |
| Mahalaxmi | 479 (83.0%) | **0.044** |
| Godawari | 487 (78.4%) |  |
| Presence of other communicable diseases in the household | | |
| Mahalaxmi | 565 (97.4%) | 0.970 |
| Godawari | 611 (97.4%) |  |
| Presence of chronic illness in the household | | |
| Mahalaxmi | 304 (52.5%) | 0.341 |
| Godawari | 311 (49.8%) |  |
| No disease present in the household |  |  |
| Mahalaxmi | 388 (66.9%) | **<0.001** |
| Godawari | 477 (67.2%) |  |
| Presence of health worker at household | | |
| Mahalaxmi | 492 (84.8%) | **0.032** |
| Godawari | 558 (89.0%) |  |

**Table 2. Responses to knowledge statements among respondents from the two municipalities.**

| **Variable** | **Yes (n, %)** | **p-value** |
| --- | --- | --- |
| Have you ever heard of a type of medicine called antibiotics? (n, %) | | |
| Mahalaxmi | 506 (87.2%) | 0.154 |
| Godawari | 74 (12.8%) |  |
| Is paracetamol an antibiotic? (n, %) | | |
| Mahalaxmi | 118 (18.6%) | 0.884 |
| Godawari | 114 (18.2%) |  |
| Is medicine used for gastric an antibiotic? (n, %) | | |
| Mahalaxmi | 78 (13.4%) | 0.988 |
| Godawari | 84 (13.4%) |  |
| Can antibiotics kill good bacteria present in our bodies? (n, %) | | |
| Mahalaxmi | 331 (57.2%) | 0.424 |
| Godawari | 343 (54.9%) |  |
| Can antibiotics cause secondary infections after killing good bacteria present in our bodies? | | |
| Mahalaxmi | 212 (36.6%) | **<0.001** |
| Godawari | 169 (27.0%) |  |
| I can recognize antibiotics in my prescription as I always differentiate antibiotics from other medicines. (n, %) | | |
| Mahalaxmi | 217 (37.4%) | **<0.001** |
| Godawari | 143 (22.8%) |  |
| Respiratory and Urinary Tract Infections are only treated by antibiotics. (n, %) | | |
| Mahalaxmi | 206 (36.6%) | 0.755 |
| Godawari | 217 (34.7%) |  |
| Antibiotics such as penicillin can cause allergic reactions if not checked with the patient with the test dose. (n, %) | | |
| Mahalaxmi | 334 (57.4%) | 0.074 |
| Godawari | 329 (52.6%) |  |
| Household storage of antibiotics for future illness can develop antibiotic resistance. (n, %) | | |
| Mahalaxmi | 230 (39.8%) | **<0.001** |
| Godawari | 181 (29.0%) |  |
| Sharing of antibiotics in household members can develop antibiotic resistance. (n, %) | | |
| Mahalaxmi | 170 (29.4%) | 0.865 |
| Godawari | 186 (29.8%) |  |
| Self-medication with antibiotics can be one of the reasons for antibiotic resistance. (n, %) | | |
| Mahalaxmi | 211 (36.5%) | 0.171 |
| Godawari | 204 (32.7%) |  |
| Antimicrobials are any medicament used to kill or inhibit growth of bacteria. (n, %) | | |
| Mahalaxmi | 198 (34.3%) | **0.012** |
| Godawari | 172 (27.6%) |  |
| If antimicrobials are taken frequently, it may stop working in the future. (n, %) | | |
| Mahalaxmi | 315 (54.5%) | **<0.001** |
| Godawari | 233 (37.3%) |  |
| Did you come across the term antibiotic/antimicrobial resistance? (n, %) | | |
| Mahalaxmi | 71 (12.3%) | 0.144 |
| Godawari | 59 (9.7%) |  |
| Antibiotic resistance is an important and serious public health problem in the world. (n, %) | | |
| Mahalaxmi | 222 (38.5%) | **<0.001** |
| Godawari | 172 (27.7%) |  |
| Acute diarrhea can be treated with antibiotics. (n, %) | | |
| Mahalaxmi | 137 (23.7%) | 0.134 |
| Godawari | 126 (20.1%) |  |
| Patients can stop taking antibiotics when their symptoms improve. (n, %) | | |
| Mahalaxmi | 262 (45.4%) | 0.415 |
| Godawari | 298 (47.8%) |  |
| Common cold can be treated with antibiotics (n, %) | | |
| Mahalaxmi | 201 (34.8%) | **<0.001** |
| Godawari | 163 (26.1%) |  |
| Antibiotics are used to reduce pain. (n, %) | | |
| Mahalaxmi | 274 (47.4%) | **0.005** |
| Godawari | 245 (39.3%) |  |
| Antibiotics can cause side effects (allergies, diarrhea, vomiting). (n, %) | | |
| Mahalaxmi | 340 (58.9%) | **<0.001** |
| Godawari | 302 (48.3%) |  |
| Antibiotic resistance is the loss of sensitivity of antibiotic to a specific bacterium. (n, %) | | |
| Mahalaxmi | 192 (33.4%) | **<0.001** |
| Godawari | 134 (21.4%) |  |

**Table 3. Attitude scores among respondents from the two municipalities**

| **Variable** | **Strongly agree** | **Agree** | **Neutral** | **Disagree** | **Strongly disagree** | **P value** |
| --- | --- | --- | --- | --- | --- | --- |
| Antimicrobial resistance has become a serious issue all over the globe (n, %) | | | | | | |
| Mahalaxmi | 16 (2.8%) | 254 (43.9%) | 273 (47.2%) | 34 (5.9%) | 1 (0.2%) | **<0.001** |
| Godawari | 1 (0.2%) | 197 (31.4%) | 274 (43.7%) |  | 2 (0.3%) |  |
| It is the responsibility of pharmacists to educate the patient on proper use of antimicrobials (n, %) | | | | | | |
| Mahalaxmi | 19 (3.3%) | 310 (53.7%) | 175 (30.3%) | 71 (12.3%) | 2 (0.3%) | **<0.001** |
| Godawari | 18 (2.9%) | 264 (42.2%) | 170 (27.2%) | 173 (27.6%) | 1 (0.1%) |  |
| New antibiotic development can solve antimicrobial resistance issue (n, %) | | | | | | |
| Mahalaxmi | 6 (1.0%) | 140 (24.3%) | 356 (61.9%) | 72 (12.5%) | 1 (0.2%) | **<0.001** |
| Godawari | 2 (0.3%) | 108 (17.3%) | 364 (58.2%) | 148 (23.7%) | 3 (0.5%) |  |
| Antibiotic can be dispensed without prescription (n, %) | | | | | | |
| Mahalaxmi | 9 (1.6%) | 104 (18.0%) | 89 (15.4%) | 272 (47.1%) | 104 (18.0%) | **0.002** |
| Godawari | 4 (0.6%) | 67 (10.7%) | 92 (14.7%) | 339 (54.3%) | 122 (19.6%) |  |
| Patients should be requested to consult a physician before dispensing an antibiotic without prescription (n, %) | | | | | | |
| Mahalaxmi | 108 (18.7%) | 332 (57.4%) | 85 (14.7%) | 53 (9.2%) | 0 (0.0%) | **<0.001** |
| Godawari | 38 (6.1%) | 388 (61.9%) | 94 (15.0%) | 106 (16.9%) | 1 (0.2%) |  |
| Patients with minor infections need not consult a physician for an antibiotic (n, %) | | | | | | |
| Mahalaxmi | 14 (2.4%) | 304 (52.7%) | 114 (19.8%) | 140 (24.3%) | 5 (0.9%) | 0.008 |
| Godawari | 3 (0.5%) | 333 (53.5%) | 102 (16.4%) | 182 (29.2%) | 3 (0.5%) |  |
| Patients with minor infections can be dispensed without prescription by pharmacists (n, %) | | | | | | |
| Mahalaxmi | 11 (1.9%) | 315 (54.5%) | 130 (22.5%) | 120 (20.8%) | 2 (0.3%) | **0.004** |
| Godawari | 3 (0.5%) | 354 (56.7%) | 103 (16.5%) | 161 (25.8%) | 3 (0.5%) |  |
| Tackling antibiotic resistance is solely the responsibility of physician (n, %) | | | | | | |
| Mahalaxmi | 11 (1.9%) | 202 (35.0%) | 256 (44.4%) | 103 (17.9%) | 5 (0.9%) | 0.007 |
| Godawari | 1 (0.2%) | 217 (34.3%) | 257 (41.1%) | 142 (22.7%) | 9 (1.4%) |  |
| Reasons for dispensing antibiotics without prescription maybe the business benefit of store (n, %) | | | | | | |
| Mahalaxmi | 33 (5.7%) | 327 (56.8%) | 140 (24.3%) | 75 (13.0%) | 1 (0.2%) | **<0.001** |
| Godawari | 19 (3.0%) | 338 (54.0%) | 120 (19.2%) | 147 (23.5%) | 2 (0.3%) |  |
| Reasons for dispensing antibiotics without prescription maybe no time and budget (n, %) | | | | | | |
| Mahalaxmi | 12 (2.1%) | 331 (57.4%) | 155 (26.9%) | 79 (13.7%) | 0 (0.0%) | **<0.001** |
| Godawari | 16 (2.6%) | 333 (53.2%) | 124 (19.8%) | 152 (24.3%) | 1 (0.2%) |  |
| Reasons for dispensing antibiotics without prescription (n, %) | | | | | | |
| Mahalaxmi | 7 (1.2%) | 192 (33.3%) | 289 (50.2%) | 88 (15.3%) | 0 (0.0%) | **<0.001** |
| Godawari | 12 (1.9%) | 255 (40.9%) | 195 (31.3%) | 160 (25.7%) | 1 (0.2%) |  |
| Reasons for dispensing antibiotics without prescription maybe the patient’s requests for antibiotics (n, %) | | | | | | |
| Mahalaxmi | 11 (1.9%) | 250 (43.3%) | 224 (38.8%) | 91 (15.8%) | 1 (0.2%) | **<0.001** |
| Godawari | 6 (1.0%) | 300 (48.1%) | 167 (26.8%) | 149 (23.9%) | 2 (0.3%) |  |

**Table 4. Frequency of carrying out different actions related to antibiotics among respondents in the two municipalities**

| **Variable** | Never | Rarely | Sometimes | Often | Always | **P value** |
| --- | --- | --- | --- | --- | --- | --- |
| Have you ever educated someone on when and how to use the antibiotic? (n, %) | | | | | | |
| Mahalaxmi | 364 (63.0%) | 14 (2.4%) | 146 (25.3%) | 39 (6.7%) | 15 (2.6%) | **<0.001** |
| Godawari | 358 (57.1%) | 5 (0.8%) | 120 (19.1%) | 53 (8.5%) | 91 (14.5%) |  |
| Have you educated the patient on minor side effects of antibiotics (n, %) | | | | | | |
| Mahalaxmi | 462 (80.1%) | 10 (1.7%) | 93 (16.1%) | 10 (1.7%) | 2 (0.3%) | 0.047 |
| Godawari | 535 (85.5%) | 8 (1.3%) | 79 (12.6%) | 4 (0.6%) | 0 (0.0%) |  |
| Have you ever dispensed antibiotics to known patients and friends without prescription? (n, %) | | | | | | |
| Mahalaxmi | 547 (94.6%) | 3 (0.5%) | 26 (4.5%) | 2 (0.3%) | 0 (0.0%) | 0.250 |
| Godawari | 592 (94.7%) | 8 (1.3%) | 20 (3.2%) | 5 (0.8%) | 0 (0.0%) |  |
| Have you ever used antibiotics to treat minor ailments in patient without prescription? (n, %) | | | | | | |
| Mahalaxmi | 500 (86.5%) | 9 (1.6%) | 62 (10.7%) | 6 (1.0%) | 1 (0.2%) | **<0.001** |
| Godawari | 581 (92.7%) | 18 (2.9%) | 23 (3.7%) | 5 (0.8%) | 0 (0.0%) |  |
| Do you counsel or inform the patients that misuse of antibiotics can lead to antibiotic resistance? (n, %) | | | | | | |
| Mahalaxmi | 459 (79.5%) | 13 (2.3%) | 88 (15.3%) | 16 (2.8%) | 1 (0.2%) | 0.265 |
| Godawari | 524 (84.0%) | 10 (1.6%) | 71 (11.4%) | 17 (2.7%) | 2 (0.3%) |  |

**Table 5. Answers to statements on adherence to antibiotics among respondents in the two municipalities.**

| **Variable** | **Yes (n, %)** | **p-value** |
| --- | --- | --- |
| Do you ever forget to take your antibiotics? (n, %) | | |
| Mahalaxmi | 203 (35.1%) | 0.035 |
| Godawari | 376 (64.9%) |  |
| Are you careless at times about taking your antibiotics? (n, %) | | |
| Mahalaxmi | 56 (9.7%) | 0.736 |
| Godawari | 57 (9.1%) |  |
| When you feel better, do you sometimes stop taking your antibiotics? (n, %) | | |
| Mahalaxmi | 118 (20.4%) | 0.911 |
| Godawari | 129 (20.6%) |  |
| Sometimes if you feel worse when you take the antibiotics, do you stop taking it? (n, %) | | |
| Mahalaxmi | 189 (32.7%) | **<0.001** |
| Godawari | 142 (22.7%) |  |
| I take my antibiotics only when I am sick. (n, %) |  |  |
| Mahalaxmi | 115 (19.9%) | **<0.001** |
| Godawari | 186 (29.9%) |  |
| It is unnatural for my mind and body to be controlled by antibiotics. (n, %) | | |
| Mahalaxmi | 292 (50.5%) | 0.050 |
| Godawari | 280 (44.9%) |  |
| My thoughts are clearer on antibiotics (n, %) | | |
| Mahalaxmi | 395 (68.6%) | 0.077 |
| Godawari | 459 (73.2%) |  |
| By staying on antibiotics, I can prevent getting sick (n, %) | | |
| Mahalaxmi | 198 (34.2%) | 0.873 |
| Godawari | 211 (33.8%) |  |
| I feel weird like a ‘zombie’ on antibiotics (n, %) | | |
| Mahalaxmi | 368 (63.6%) | 0.185 |
| Godawari | 374 (59.8%) |  |
| Antibiotics makes me feel tired and sluggish (n, %) |  |  |
| Mahalaxmi | 401 (69.4%) | 0.592 |
| Godawari | 426 (67.9%) |  |
| Overall result (n, %) | | |
| Adherence | 265 (43.6%) | **<0.001** |
| Nonadherence | 267 (46.3%) |  |

**Table 6. Knowledge and attitude scores among different subgroups of respondents (in the two municipalities surveyed)**

| **Characteristic** | **Knowledge score**  **Mean (±SD)** | **P value** | **Attitude score Median (IQR)** | **P value** |
| --- | --- | --- | --- | --- |
| Highest education | | | | |
| No formal education | 8.80 ± 2.6 | <0.001 | 38 (7) | **0.003** |
| Primary | 9.88 ± 2.8 |  | 42 (6) |  |
| Secondary | 10.33 ± 2.78 |  | 41 (8) |  |
| Higher secondary | 11.39 ± 2.6 |  | 40 (8) |  |
| Bachelor’s degree and above | 11.62 ± 2.8 |  | 40 (8) |  |
| Occupation | | | | |
| No work | 9.95 ± 3.03 | <0.001 | 42 (9) | **<0.001** |
| Daily wage | 9.98 ± 2.04 |  | 39 (7) |  |
| Retired | 11.02 ± 2.94 |  | 41 (12) |  |
| Home maker | 9.40 ± 2.79 |  | 40 (7) |  |
| Other | 9.47 ± 3.21 |  | 40 (7) |  |
| Presence of respiratory disease in the household | | | | |
| Yes | 9.70 ± 2.90 | 0.414 | 39 (9) | 0.027 |
| No | 9.88 ± 2.81 |  | 40 (8) |  |
| Presence of other communicable diseases in the household | | | | |
| Yes | 9.74 ± 2.90 | 0.596 | 39 (12) | 0.068 |
| No | 9.48 ± 2.51 |  | 40 (8) |  |
| Presence of chronic diseases in the household | | | | |
| Yes | 9.91 ± 2.96 | 0.041 | 40 (8) | 0.279 |
| No | 9.55 ± 2.81 |  | 40 (8) |  |
| Presence of health worker in the household | | | | |
|  | 9.64 ± 2.85 | 0.009 | 39 (12) | 0.068 |
|  | 10.32 ± 3.09 |  | 40 (8) |  |

Table 7. Practice and adherence scores among different subgroups of respondents (in the two municipalities surveyed)

| **Characteristic** | **Practice score Median (IQR)** | **P value** | **Adherence score Median (IQR)** | **P value** |
| --- | --- | --- | --- | --- |
| Highest Education |  |  |  |  |
| No formal education | 5 (2) | **<0.001** | 6 (3) | **<0.001** |
| Primary | 5 (4) |  | 5 (3) |  |
| Secondary | 7 (4) |  | 5 (3) |  |
| Higher secondary | 9 (6) |  | 6 (3) |  |
| Bachelor’s degree and above | 8 (5) |  | 6 (2) |  |
| Occupation |  |  |  |  |
| No work | 5 (2) | **<0.001** | 6 (3) | **<0.001** |
| Daily wage | 5 (4) |  | 5.5 (2) |  |
| Retired | 7 (4) |  | 6 (2) |  |
| Home maker | 7 (4) |  | 5 (3) |  |
| Other | 8 (4) |  | 5 (3) |  |
| Presence of respiratory disease in the household | | | | |
| Yes | 5 (4) | 0.249 | 5 (3) | 0.249 |
| No | 6 (4) |  | 6 (3) |  |
| Presence of other communicable diseases in the household | | | | |
| Yes | 5 (4) | 0.418 | 6 (3) | 0.418 |
| No | 6 (4) |  | 6 (3) |  |
| Presence of chronic diseases in the household | | | | |
| Yes | 6 (4) | .0377 | 5 (3) | 0.377 |
| No | 6 (4) |  | 6 (3) |  |
| Presence of health worker in the household | | | | |
| Yes | 7 (4) | 0.206 | 6 (3) | 0.206 |
| No | 5 (4) |  | 6 (3) |  |
