# Supplementary material for: Baseline assessment of knowledge, attitude, practice, and adherence toward antimicrobials among women living in two urban municipalities in Lalitpur district, Nepal
Source: PLoS One. 2025 Jan 9;20(1):e0317092. doi: 10.1371/journal.pone.0317092 (PMC11717222; doi:10.1371/journal.pone.0317092)
Supplement: S1 Questionnaire — (DOCX) [file pone.0317092.s003.docx]

**Questionnaire for Baseline Survey**

Title for Research: Development, validation and evaluation of a collaborative educational intervention program on antimicrobial use and compliance in selected urban areas of Lalitpur district.

**Section-1: Demographics**

| Age (years):                     Gender: Female/Male                       District:  Able to read and write: Yes/No  Highest Education Level: a. No formal education b. Primary c. Secondary d. Higher Secondary e. Bachelors f. Masters g. Above than Masters  Occupation: a. No work b. Daily wage labor c. Service d. Retired e. Home maker f. Others ………….  Work Experience (years or months):………………..  Average Monthly Income in NPR:  a. No income b. Below 20,000 c. 20,000–40,000 d. 40,000–60,000 e. 60,000–80,000 f. 80,000 and above  Number of household members: ….  Total number of household members with chronic disease:...  Presence of infectious/other disease:  a.     Respiratory tract infection: Yes/No  b.     Other infectious diseases (TB): Yes/No  c.     Chronic illness (Hypertension, Diabetes): Yes/No  d. No disease: Yes/No  Presence of healthcare professional at home: Yes/No |
| --- |

**Section-2: Knowledge on antimicrobials and antimicrobial resistance**

| 1. Knowledge regarding the identification of antibiotics   1. Have you ever heard of a type of medicine called antibiotics? Yes/No 2. Is amoxicillin an antibiotic? Yes/No 3. Is paracetamol an antibiotic? Yes/No 4. Is medicine used for gastric an antibiotic? Yes/No   2. Knowledge regarding the uses of antibiotics   1. Can antibiotics kill good bacteria present in our bodies? Yes/No 2. Can antibiotics cause secondary infections after killing good bacteria present in our bodies? Yes/No   3. I can recognize antibiotics in my prescription as I always differentiate antibiotics from other medicines.  Yes/No  4. Respiratory Tract Infections and Urinary Tract Infections (UTIs) are only treated by antibiotics.  Yes/No  5. Antibiotics such as penicillins can cause allergic reactions if not checked with the patient with the test dose.  Yes/No  6. Household storage of antibiotics for future illness can develop antibiotic resistance.  Yes/No  7. Sharing of antibiotics in household members can develop antibiotic resistance.  Yes/No  8. Self-medication with antibiotics can be one of the reasons for antibiotic resistance.  Yes/No  9.   Antimicrobials are any medicament used to kill or inhibit growth of bacteria. Yes/No  10.     If antimicrobials are taken frequently, it may stop working in the future. Yes/No  11.     Did you come across the term antibiotic/antimicrobial resistance? Yes/No  12.     Antibiotic resistance is an important and serious public health problem in the world. Yes/No  13.     Acute diarrhea can be treated with antibiotics. Yes/No  14.     Patients can stop taking antibiotics when their symptoms improve before the full antibiotic course.  Yes/No  15.     Common cold can be treated with antibiotics. Yes/No  16.     Antibiotics are used to reduce pain. Yes/No  17.     Antibiotics can cause side effects (allergies, diarrhea, vomiting). Yes/No  18.  Antibiotic resistance is the loss of sensitivity of antibiotic to a specific bacterium. Yes/No |
| --- |

**Section-3: Attitudes towards antibiotic resistance**

| 1.     Antimicrobial resistance has become a serious issue all over the globe.   1. Strongly agree b. Agree c. Neutral d. Disagree e. Strongly disagree   2.     It is the responsibility of pharmacists to educate the patient on proper use of antimicrobials.   1. Strongly agree b. Agree c. Neutral d. Disagree e. Strongly disagree   3.     New antibiotic development can solve antimicrobial resistance issue   1. Strongly agree b. Agree c. Neutral d. Disagree e. Strongly disagree   4.     Antibiotic can be dispensed without prescription.   1. Strongly agree b. Agree c. Neutral d. Disagree e. Strongly disagree   5.     Patients should be requested to consult a physician before dispensing an antibiotic without prescription.   1. Strongly agree b. Agree c. Neutral d. Disagree e. Strongly disagree   6.     Patients with minor infections (pharyngitis, diarrhea etc.) need not consult a physician for an antibiotic, it can be dispensed without prescription by pharmacists.   1. Strongly agree b. Agree c. Neutral d. Disagree e. Strongly disagree   7.     Patients with minor infections (pharyngitis, diarrhea etc.) can be dispensed without prescription by pharmacists.   1. Strongly agree b. Agree c. Neutral d. Disagree e. Strongly disagree   8.     Tackling antibiotic resistance is solely the responsibility of physician.   1. Strongly agree b. Agree c. Neutral d. Disagree e. Strongly disagree   9. Reasons for dispensing antibiotics without prescription maybe the business benefit of store.   1. Strongly agree b. Agree c. Neutral d. Disagree e. Strongly disagree   10. Reasons for dispensing antibiotics without prescription maybe no time and budget to visit physicians by the patients..   1. Strongly agree b. Agree c. Neutral d. Disagree e. Strongly disagree   11. Reasons for dispensing antibiotics without prescription maybe the competency of community pharmacists to treat common infections.   1. Strongly agree b. Agree c. Neutral d. Disagree e. Strongly disagree   12. Reasons for dispensing antibiotics without prescription maybe the patient’s requests for antibiotics.   1. Strongly agree b. Agree c. Neutral d. Disagree e. Strongly disagree |
| --- |

**Section-4: Practice for use of antibiotics**

| 1. Have you ever educated someone on when and how to use the antibiotic?   1. Never b. Rarely c. Sometimes d. Often e. Always   2. Have you educated the patient on minor side effects of antibiotics?   1. Never b. Rarely c. Sometimes d. Often e. Always   3. Have you ever dispensed antibiotics to known patients and friends without prescription?   1. Never b. Rarely c. Sometimes d. Often e. Always   4. Have you ever used antibiotics to treat minor ailments in patient without prescription?   1. Never b. Rarely c. Sometimes d. Often e. Always   5. Do you counsel or inform the patients that misuse of antibiotics can lead to antibiotic resistance?   1. Never b. Rarely c. Sometimes d. Often e. Always |
| --- |

**Section-5: Questionnaire assessing adherence to antibiotic therapy**

| 1. Do you ever forget to take your antibiotics? Yes/No  2. Are you careless at times about taking your antibiotics? Yes/No  3. When you feel better, do you sometimes stop taking your antibiotics? Yes/No  4. Sometimes if you feel worse when you take the antibiotics, do you stop taking it? Yes/No  5. I take my antibiotics only when I am sick. Yes/No  6. It is unnatural for my mind and body to be controlled by antibiotics.? Yes/No  7. My thoughts are clearer on antibiotics. Yes/No  8. By staying on antibiotics, I can prevent getting sick. Yes No  9. I feel weird like a ‘zombie’ on antibiotics. Yes No  10. Antibiotics makes me feel tired and sluggish. Yes no |
| --- |

*** Thank you ***
