# Supplementary material for: Baseline assessment of knowledge, attitude, practice, and adherence toward antimicrobials among women living in two urban municipalities in Lalitpur district, Nepal
Source: PLoS One. 2025 Jan 9;20(1):e0317092. doi: 10.1371/journal.pone.0317092 (PMC11717222; doi:10.1371/journal.pone.0317092)
Supplement: S1 Checklist — (DOC) [file pone.0317092.s004.doc]

STROBE Statement—Checklist of items that should be included in reports of ***cross-sectional studies***

|  | Item No | Recommendation |
| --- | --- | --- |
| **Title and abstract** | 1 | 1. Indicate the study’s design with a commonly used term in the title or the abstract   **Study design: quasi-experimental design (Page no.: 2, abstract; page number 4, Methods section)** |
|  |  | 1. Provide in the abstract an informative and balanced summary of what was done and what was found   **Summary: We tried to assess mother’s groups KAP and adherence towards antimicrobial use and resistance resistance using a quasi-experimental design where Mahalaxmi municipality is the intervention and Godavari is the comparison/control area. The study population was women belonging to the mother’s groups of 45 female community health volunteers (FCHVs) from each of these municipalities. Knowledge, attitude, practice and adherence scores among different subgroups of respondents in the two municipalities were found to be significantly different for occupation, and education**.  **(Page no.: 2)** |
| Introduction | | |
| Background/rationale | 2 | Explain the scientific background and rationale for the investigation being reported  **Community engagement can be important for involving people from the community towards understanding the problem of AMR and the ways to prevent it. As per a 2017 definition, community engagement is a participatory approach for identification, developing and implementing community led sustainable interventions for problems of their concern. Engaging the community towards reducing the problem of AMR can be an important approach. The national antimicrobial resistance containment action plan of Nepal was developed in 2016 and mentions AMR as one of the national priorities. The plan mentions AMR as a shared responsibility for all stakeholders. Resistant species of microbes can also be a reason for the easy spread of AMR in communities via contaminated food and water. Direct contact with people, animals and the environment can also be a reason for the development of AMR. The present study is being done in a community as this may be the right place to educate and communicate about the responsible use of antimicrobials and the burden due to AMR. (Page no.: 3)** |
| Objectives | 3 | State specific objectives, including any prespecified hypotheses  **The aim of the study was to assess the knowledge, attitude, practice, and adherence toward antimicrobials among women living in two urban municipalities in Lalitpur district, Nepal. (Page no.: 4)** |
| Methods | | |
| Study design | 4 | Present key elements of study design early in the paper  **A quasi-experimental study is being conducted in two urban municipalities of Lalitpur district, Mahalaxmi (intervention) and Godavari (comparison). This was a questionnaire-based survey for collecting baseline information for the quasi-experimental study. (Page no.: 4, Method section)** |
| Setting | 5 | Describe the setting, locations, and relevant dates, including periods of recruitment, exposure, follow-up, and data collection  **Data was collected from all women belonging to the mother’s group of FCHVs of intervention and comparison areas during September 2023 to January 2024. Ethical approval for the quasi-experimental study was obtained by the Ethical review board of Nepal Health Research Council on 29^th^ August 2023 including approval for the baseline study with a reference number 296 and protocol registration number 535/2023. Informed consent was obtained in written form from each participant prior to the start of the study. All the ethical principles laid down by the ethical approving body were followed strictly.(Page no.: 4,5 Method section)** |
| Participants | 6 | 1. Give the eligibility criteria, and the sources and methods of selection of participants   **The women belonging to the mother’s group of 45 FCHVs in each of the two municipalities were the study population who have provided informed consent to participate in the study were selected for the study. (Page no.: 4, 5 and 6)** |
| Variables | 7 | Clearly define all outcomes, exposures, predictors, potential confounders, and effect modifiers. Give diagnostic criteria, if applicable  **The outcomes we were measuring were mothers knowledge, attitude, practice and adherence towards antimicrobial use and resistance**  **A variety of factors could impact these including the level of education of the mothers, their work experience and their location.** |
| Data sources/ measurement | 8* | For each variable of interest, give sources of data and details of methods of assessment (measurement). Describe comparability of assessment methods if there is more than one group  **The variables of interest were measured using a questionnaire developed from a previous studies. The data was collected by trained data collectors who visited the mothers in the two municipalities where they were residing. (page 6,7).** |
| Bias | 9 | Describe any efforts to address potential sources of bias  **The questionnaire was validated for face and content validation. A census sample of all mothers belonging to the FCHVs was chosen. The data was collected using KOBO Toolbox in android tablets by the research team and trained data collectors. (page 6,7).** |
| Study size | 10 | Explain how the study size was arrived at  **The sample size was 1207 mothers, 580 mothers in Mahalaxmi and 627 mothers in Godawari municipalities. This was calculated based on the prevalence of adherence to antibiotics of 39.1% in an Ethiopian study. (page 5).** |
| Quantitative variables | 11 | Explain how quantitative variables were handled in the analyses. If applicable, describe which groupings were chosen and why  **Descriptive analysis was done for the quantitative variables** |
| Statistical methods | 12 | 1. Describe all statistical methods, including those used to control for confounding   **Descriptive analysis was done** |
|  |  | 1. Describe any methods used to examine subgroups and interactions   **Yes, subgroup analysis was performed using appropriate parametric and non parametric tests as appropriate.** |
|  |  | 1. Explain how missing data were addressed   N/A |
|  |  | 1. If applicable, describe analytical methods taking account of sampling strategy   N/A |
|  |  | 1. Describe any sensitivity analyses   N/A |
| Results | | |
| Participants | 13* | 1. Report numbers of individuals at each stage of study—eg numbers potentially eligible, examined for eligibility, confirmed eligible, included in the study, completing follow-up, and analysed   **This is mentioned on pages 5 and 6.** |
|  |  | 1. Give reasons for non-participation at each stage   **We did not measure non-participation in the study. We approached participants for participation and if they agreed and met the inclusion criteria we included them in the study.** |
|  |  | 1. Consider use of a flow diagram   **Due to the reasons mentioned in b above a flow diagram may not be applicable in this study.** |
| Descriptive data | 14* | 1. Give characteristics of study participants (eg demographic, clinical, social) and information on exposures and potential confounders   **This is mentioned on page 7 and Table 1.** |
|  |  | 1. Indicate number of participants with missing data for each variable of interest   **We did not have missing data.** |
| Outcome data | 15* | Report numbers of outcome events or summary measures  **Mentioned in Results section and Tables (pages 7 to 18).** |
| Main results | 16 | 1. Give unadjusted estimates and, if applicable, confounder-adjusted estimates and their precision (eg, 95% confidence interval). Make clear which confounders were adjusted for and why they were included   NA |
|  |  | 1. Report category boundaries when continuous variables were categorized   **This has been mentioned in the Results section.** |
|  |  | 1. If relevant, consider translating estimates of relative risk into absolute risk for a meaningful time period   **NA** |
| Other analyses | 17 | Report other analyses done—eg analyses of subgroups and interactions, and sensitivity analyses  **NA** |
| Discussion | | |
| Key results | 18 | Summarise key results with reference to study objectives  **Knowledge was higher in Mahalaxmi municipality, but adherence was higher in Godawari municipality (p <0.0001), but no significant difference was seen in attitude and practice scales. Knowledge, attitude, practice and adherence scores among different subgroups of respondents in the two municipalities were found to be significantly different for occupation (p <0.0001), and education (p <0.0001). The attitude scores were also significantly different according to presence/absence of respiratory disease in the household (p = 0.027). (Page 11, 16)** |
| Limitations | 19 | Discuss limitations of the study, taking into account sources of potential bias or imprecision. Discuss both direction and magnitude of any potential bias  **The major limitation of this study is that it has been conducted only in two municipalities of Lalitpur district. The study design of this study is quasi experimental and thus cannot represent the results of experimental study resign. The sampling method is not random sampling hence the result may not be generalizable. Studies have shown that there is also a role of community pharmacy people in dispensing antimicrobials without prescription. No intervention for the community pharmacies in this research project is another limitation. (Page 23)** |
| Interpretation | 20 | Give a cautious overall interpretation of results considering objectives, limitations, multiplicity of analyses, results from similar studies, and other relevant evidence  **Has been discussed in the discussion section.** |
| Generalisability | 21 | Discuss the generalisability (external validity) of the study results  **The study was only done in two urban municipalities in one district of the Kathmandu valley. Hence the study findings may not be generalizable to other districts and to rural areas outside the valley. The external validity of our findings may be limited (mentioned on page 23)** |
| Other information | | |
| Funding | 22 | Give the source of funding and the role of the funders for the present study and, if applicable, for the original study on which the present article is based  **Funded by University Grants Commission, collaborative research grant (CRG-79/80-HS-01). The funder did not influence the data collection procedures, the analysis and the interpretation of the findings.** |

*Give information separately for exposed and unexposed groups.

**Note:** An Explanation and Elaboration article discusses each checklist item and gives methodological background and published examples of transparent reporting. The STROBE checklist is best used in conjunction with this article (freely available on the Web sites of PLoS Medicine at http://www.plosmedicine.org/, Annals of Internal Medicine at http://www.annals.org/, and Epidemiology at http://www.epidem.com/). Information on the STROBE Initiative is available at www.strobe-statement.org.
