# Supplementary material for: Baseline assessment of knowledge, attitude, practice, and adherence toward antimicrobials among women living in two urban municipalities in Lalitpur district, Nepal
Source: PLoS One. 2025 Jan 9;20(1):e0317092. doi: 10.1371/journal.pone.0317092 (PMC11717222; doi:10.1371/journal.pone.0317092)
Supplement: Attachment — Submitted filename: Revision letter 27th Nov.docx [file pone.0317092.s007.docx]

**Revision letter**

Lalitpur, Nepal

Date: 27^th^ November, 2024

To

The Editor-in-Chief

PLOS ONE

Sub: Submission of the revised version of the manuscript for consideration of publication

Dear Editor-in-Chief,

We are resubmitting the manuscript “PONE-D-24-31683 - Knowledge, attitude, practice, and adherence toward antimicrobials among women living in two urban municipalities in Lalitpur district, Nepal” after revising it and making necessary corrections for consideration of publication in your esteemed journal. We remain grateful to the reviewers for their constructive comments.

The response to specific comments is as follows:

**General comments**

Comment for Journal requirements:

When submitting your revision, we need you to address these additional requirements.

1. Please ensure that your manuscript meets PLOS ONE's style requirements, including those for file naming. The PLOS ONE style templates can be found at

[https://journals.plos.org/plosone/s/file?id=wjVg/PLOSOne_formatting_sample_main_body.pdf](https://journals.plos.org/plosone/s/file?id=wjVg/PLOSOne_formatting_sample_main_body.pdf#inbox/_blank) and

[https://journals.plos.org/plosone/s/file?id=ba62/PLOSOne_formatting_sample_title_authors_affiliations.pdf](https://journals.plos.org/plosone/s/file?id=ba62/PLOSOne_formatting_sample_title_authors_affiliations.pdf#inbox/_blank)

Response: Thank you. The manuscript has been modified to meet the journal criteria.

Comment-2. Thank you for stating the following financial disclosure:

“University Grants Commission collaborative research grant. Grant number CRG-79/80-HS-01.”

Please state what role the funders took in the study.  If the funders had no role, please state: "The funders had no role in study design, data collection and analysis, decision to publish, or preparation of the manuscript."

Response: The funders had no role in the study design, data collection and analysis, decision to publish, or preparation of the manuscript.

If this statement is not correct you must amend it as needed.

Please include this amended Role of Funder statement in your cover letter; we will change the online submission form on your behalf.

Comment-3. Thank you for stating the following in the Competing Interests section:

“All authors have no conflict of interest except one author, Pathiyil Ravi Shankar, who is an academic editor at PLoS One.”

Please confirm that this does not alter your adherence to all PLOS ONE policies on sharing data and materials, by including the following statement: "This does not alter our adherence to  PLOS ONE policies on sharing data and materials.” (as detailed online in our guide for authors [http://journals.plos.org/plosone/s/competing-interests](http://journals.plos.org/plosone/s/competing-interests#inbox/_blank)).  If there are restrictions on sharing of data and/or materials, please state these. Please note that we cannot proceed with consideration of your article until this information has been declared.

Please include your updated Competing Interests statement in your cover letter; we will change the online submission form on your behalf.

Response: We have included the statement ‘This does not alter our adherence to PLOS ONE policies on sharing data and materials’.

Comment -4. Thank you for stating the following in the Acknowledgments Section of your manuscript:

“Authors would like to thank all the participants from both the municipalities. We also like to thank the ward chairs of the Godavari and Mahalaxmi municipalities for allowing us to conduct the research in their areas. We also thank the University Grants Commission for grants us with the collaborative research grant with the grant number CRG-79/80-HS-01.”

We note that you have provided funding information that is currently declared in your Funding Statement. However, funding information should not appear in the Acknowledgments section or other areas of your manuscript. We will only publish funding information present in the Funding Statement section of the online submission form.

Please remove any funding-related text from the manuscript and let us know how you would like to update your Funding Statement. Currently, your Funding Statement reads as follows:

“University Grants Commission collaborative research grant. Grant number CRG-79/80-HS-01.”

Please include your amended statements within your cover letter; we will change the online submission form on your behalf.

Response: The funders information from the acknowledgement section has been deleted as suggested.

Comment -5. In the online submission form, you indicated that [The data underlying the study will be made available from the corresponding author upon reasonable request.].

All PLOS journals now require all data underlying the findings described in their manuscript to be freely available to other researchers, either 1. In a public repository, 2. Within the manuscript itself, or 3. Uploaded as supplementary information.

This policy applies to all data except where public deposition would breach compliance with the protocol approved by your research ethics board. If your data cannot be made publicly available for ethical or legal reasons (e.g., public availability would compromise patient privacy), please explain your reasons on resubmission and your exemption request will be escalated for approval.

**Response:** The data is deposited as an Excel file in Figshare. The link is Shankar, P Ravi; Jha, Nisha; Kafle, Sajala; Joshi, Mili; Pandey, Aakriti; Koirala, Prakriti; et al. (2024). Baseline data on KAP and adherence to antimicrobial therapy in two municipalities of Lalitpur district, Nepal. figshare. Dataset. <https://doi.org/10.6084/m9.figshare.27610713.v1>

Additional Editor Comments:

The intervention study is very timely and needed. Please do following revision and also go through all the comments from reviewers.
Comment 1: Check the spelling of Godawari Municipality

Response: Thank you. The spelling of Godawari municipality is correct. This is how it has been spelt in English on the government portal <http://www.godawarimun.gov.np/en>

Comment 2: Introduction part - no need to write the methodology but add the importance of the study

Response: We have mentioned why this study is important. (pages 5 and 9 in the manuscript with track changes)

Comment 3: Write details of data analysis in methodology part
Response: This has been done. (page 15).

Comment 4: Check the table percentage (whether row or column %) as you are comparing two municipalities
Response: We have rearranged the tables.

Comment 5: Revisit the table and try to make it more efficient
Response: We are only presenting the significantly different values in the tables. This we believe makes it more efficient.

Thank you so much.


Reviewers' comments:

Reviewer's Responses to Questions

**Comments to the Author**

1. Is the manuscript technically sound, and do the data support the conclusions?

The manuscript must describe a technically sound piece of scientific research with data that supports the conclusions. Experiments must have been conducted rigorously, with appropriate controls, replication, and sample sizes. The conclusions must be drawn appropriately based on the data presented.

Reviewer #1: Partly

Reviewer #2: Yes

Reviewer #3: Yes

2. Has the statistical analysis been performed appropriately and rigorously?

Reviewer #1: Yes

Reviewer #2: Yes

Reviewer #3: Yes

3. Have the authors made all data underlying the findings in their manuscript fully available?

The [PLOS Data policy](http://c05y1x9s.r.us-east-2.awstrack.me/L0/http://www.plosone.org/static/policies.action%23sharing/1/010f01921d4afbe0-3a3c4ea5-7740-44d0-96eb-e110f46d0ae2-000000/FzS0ggi1Q8ECknusDgVCVB3Ro7E=177#inbox/_blank) requires authors to make all data underlying the findings described in their manuscript fully available without restriction, with rare exception (please refer to the Data Availability Statement in the manuscript PDF file). The data should be provided as part of the manuscript or its supporting information, or deposited to a public repository. For example, in addition to summary statistics, the data points behind means, medians and variance measures should be available. If there are restrictions on publicly sharing data—e.g. participant privacy or use of data from a third party—those must be specified.

Reviewer #1: Yes

Reviewer #2: Yes

Reviewer #3: Yes

The data has been deposited on a public repository as mentioned previously.

4. Is the manuscript presented in an intelligible fashion and written in standard English?

PLOS ONE does not copyedit accepted manuscripts, so the language in submitted articles must be clear, correct, and unambiguous. Any typographical or grammatical errors should be corrected at revision, so please note any specific errors here.

Reviewer #1: No

Reviewer #2: Yes

Reviewer #3: Yes

The manuscript has been copyedited for language and grammar.

5. Review Comments to the Author

Please use the space provided to explain your answers to the questions above. You may also include additional comments for the author, including concerns about dual publication, research ethics, or publication ethics. (Please upload your review as an attachment if it exceeds 20,000 characters)

**Reviewer #1: 1. General**
Jha et al assessed KAPs on antimicrobial use in Nepal. The manuscript is potentially relevant but would require major revision to make it fit for publication. It has a lot of information but poorly organized.

We have reorganized the manuscript as suggested by the editor and the reviewers.

2. Introduction
3. a. Authors described the burden and public health importance of AMR but without statistics. Kindly include statistics at global, regional, and national levels to give weight to the problem

Response: We have addressed this on page 5.

b. What did previous studies find about AMR, knowledge, attitude, and use of antibiotics among community members? What were the gaps? Which gap(s) is the current study seeking to address?

Response: We have tried to address this on page 9.

c. Merge lines 79-81 with 97-98

Response: Has been merged as suggested.

3. Methods

a. How were the study areas classified? What differentiates the study areas as intervention and control?

Response: Mahalaxmi and Godawari Municipalities are neighboring municipalities in Lalitpur District of Nepal. The Mahalaxmi Municipality occupies 26.5 square km. The municipality has around 62 thousand population. The Godawari municipality is larger occupying an area of 96.11 sq.km with total population being around 116 thousand. There were no specific reasons for classifying one municipality as control and the other as intervention.

b. How was the data analyzed? It is important to include a brief on data analysis in this section.

Response: This has been described. (page 15)

c. More respondents were selected in Godawari municipality compared with Mahalaxmi. Why the unequal sample size?

Response: The number of FCHVs in the Godawari municipality was more. The number of mothers associated with mothers’ groups varied from 10 to 25 as mentioned in the appendix. We selected an equal number of FCHVs from both municipalities. There were more mothers associated with the selected mothers’ groups in Godawari as compared to Mahalaxmi.

Why was Lalitpur district selected for the study and not any other district in the province or country?
Response: Mainly due to convenience as the researchers were working in Lalitpur district and this made it easier to do the study there. Also, Lalitpur district is part of the Kathmandu valley and has people from all over the country.

e. Background information on the study site is inadequate. Authors should provide additional information on the health profile of the study area including access to healthcare, disease burden etc.

Response: This has been mentioned on page 6 and details of the two study areas are shown in the appendix.

4. Results

a. In lines 154-156 authors reported that presence of respiratory disease was found not to be different among the two municipalities. However, in line 156-158, presence of respiratory disease was found to differ among the two locations. Kindly clarify.

Response: Thank you for highlighting this mistake. We have corrected this as presence of respiratory disease was found to differ between the two locations.

c. The results in table 1 have not been adequately described. Authors should give a succinct description of the outputs in the table.

Response: We have added this description in addition to the existing information to provide a succinct description of the outputs in the table. (page 16)

5. Discussion

a. The discussion section appears to be an extension or repetition of the results. Authors should reorganize, explain the meaning of the results, relate them to studies done elsewhere, and reasons for any differences observed

Response: We have reworked certain areas of the discussion. We have mentioned the meaning of the results and compared these to the literature.

b. Lines 313-325 are better placed under introduction

Response: These lines has been moved as suggested.

5. Conclusion
a. A paragraph on the key findings should precede the recommendations

Response: We have drafted this as suggested (page 45).

**Reviewer #2:**

This study describes the results of a survey conducted in two districts of Nepal. The survey collected information about the awareness regarding antibiotics and antimicrobial resistance, attitude towards the use of antibiotics and adherence to treatment with antibiotics among woman in these two districts, using a structured questionnaire.
Below, please find my comments:

Comment- 1: Page 4 – Please, define the abbreviation FCHV when you first mention it in the main text of the manuscript.

Response: We have defined the term FCHV as suggested.

Comment -2: How big is the problem of AMR in Nepal? Are there any data about the resistance of specific bacterial pathogens to specific antibiotics? Please describe the situation in the country in this regard.

We have tried to answer this in the Introduction (page 5).

Response:

Comment -3: How similar the selected two districts - Mahalaxmi and Godawari - are in terms of urban vs rural areas, population demographics, prevalence of infectious disease, etc. Please, provide some information about this.

Response: Information about the two municipalities has been added as suggested.

Comment -4: Page 5, Methods – I would not characterize this study as quasi-experimental. This is a population survey and would recommend referring to it as such.

Response: We have corrected the study as a population study.

Comment -5: Page 5 – Sampling techniques: You indicate: “All women belonging to the mother’s group of FCHVs of intervention and comparison areas were included in the baseline survey” – in your assessment, how much are these participants representative of the general population of the two districts you studied?

Response: The current study is not representative at the general population level as the objective was not to generalize at that level. It is representative of the mothers belonging to the mother's group of these two municipalities. Thus, we can compare socio-demographic and other output and outcome variables among the mothers of these two municipalities. These mothers are not represented at the district level rather they are representative at the selected municipality only.

Comment -6: Page 24 – In the limitations section: “No intervention for the community pharmacies in this research project is another limitation.” – since this is a population survey, it was not required to have pharmacies participating in the study. I would not consider it as a limitation.

Response: This has been corrected as suggested.

**Reviewer #3:**

Comment-1: Would like to request a clarification in table 1 regarding the variable 'No disease present in the household' followed by yes and no requires some clarity whether yes means that there is disease or there is no disease. Also if a future study is planned as an intervention clarification regarding why respiratory illness was included as a separate illness along with other communicable diseases.

Response: No disease present in the household meant about the presence of any disease in the household and yes meant a disease was present in that household while no meant it was not present. Inclusion of respiratory illness as a separate illness was done as the use of antibiotics are commonly seen in these conditions.

Comment-2: Would also like to request the authors to add something in the title to indicate that this study is the first part of a larger study which to be done as a quasi-experimental study which would give some more clarity to the title as to why this is being done is as an initial step towards the intervention study being done at a later date.

Response: Baseline assessment of knowledge, attitude, practice, and adherence toward antimicrobials among women living in two urban municipalities in Lalitpur district, Nepal. The term baseline has been added.

6. PLOS authors have the option to publish the peer review history of their article ([what does this mean?](https://c05y1x9s.r.us-east-2.awstrack.me/L0/https://journals.plos.org/plosone/s/editorial-and-peer-review-process%23loc-peer-review-history/1/010f01921d4afbe0-3a3c4ea5-7740-44d0-96eb-e110f46d0ae2-000000/ECLIRiOx1g54Ca_71IjIu6dgN2Q=177#inbox/_blank)). If published, this will include your full peer review and any attached files.


If you choose “no”, your identity will remain anonymous but your review may still be made public.


**Do you want your identity to be public for this peer review?** For information about this choice, including consent withdrawal, please see our [Privacy Policy](https://c05y1x9s.r.us-east-2.awstrack.me/L0/https://www.plos.org/privacy-policy/1/010f01921d4afbe0-3a3c4ea5-7740-44d0-96eb-e110f46d0ae2-000000/6BysijOYXkuYcz2dLbGHQUl6LBs=177#inbox/_blank).

Reviewer #1: No

Reviewer #2: No

Reviewer #3: No

The changes have been carried out using track changes in the manuscript

Hoping for a favourable consideration

With regards

Dr. Jha and coauthors
