# Supplementary material for: Baseline assessment of knowledge, attitude, practice, and adherence toward antimicrobials among women living in two urban municipalities in Lalitpur district, Nepal
Source: PLoS One. 2025 Jan 9;20(1):e0317092. doi: 10.1371/journal.pone.0317092 (PMC11717222; doi:10.1371/journal.pone.0317092)
Supplement: Attachment — Submitted filename: REVIEWER COMMENTS.docx [file pone.0317092.s006.docx]

REVIEWER COMMENTS

1. General

Jha et al assessed KAPs on antimicrobial use in Nepal. The manuscript is potentially relevant but would require major revision to make it fit for publication. It has a lot of information but poorly organized.

2. Introduction

a. Authors described the burden and public health importance of AMR but without statistics. Kindly include statistics at global, regional, and national levels to give weight to the problem

b. What did previous studies find about AMR, knowledge, attitude, and use of antibiotics among community members? What were the gaps? Which gap(s) is the current study seeking to address?

c. Merge lines 79-81 with 97-98

3. Methods

a. How were the study areas classified? What differentiates the study areas as intervention and control?

b. How was the data analyzed? It is important to include a brief on data analysis in this section.

c. More respondents were selected in Godawari municipality compared with Mahalaxmi. Why the unequal sample size?

d. Why was Lalitpur district selected for the study and not any other district in the province or country?

e. Background information on the study site is inadequate. Authors should provide additional information on the health profile of the study area including access to healthcare, disease burden etc

4. Results

a. In lines 154-156 authors reported that presence of respiratory disease was found not to be different among the two municipalities. However, in line 156-158, presence of respiratory disease was found to differ among the two locations. Kindly clarify

b. The results in table 1 have not been adequately described. Authors should give a succinct description of the outputs in the table

5. Discussion

a. The discussion section appears to be an extension or repetition of the results. Authors should reorganize, explain the meaning of the results, relate them to studies done elsewhere, and reasons for any differences observed

b. Lines 313-325 are better placed under introduction

6. Conclusion
   a. A paragraph on the key findings should precede the recommendations
